# Membrane bound IL-21 based NK cell feeder cells drive robust expansion and metabolic activation of NK cells

**DOI:** 10.1038/s41598-019-51287-6

**Published:** 2019-10-17

**Authors:** Evelyn O. Ojo, Ashish Arunkumar Sharma, Ruifu Liu, Stephen Moreton, Mary-Ann Checkley-Luttge, Kalpana Gupta, Grace Lee, Dean A. Lee, Folashade Otegbeye, Rafick-Pierre Sekaly, Marcos de Lima, David N. Wald

**Affiliations:** 1 Department of Pathology, Case Western Reserve University, Cleveland, Ohio USA; 2 Department of Molecular Biology and Microbiology, Case Western Reserve University, Cleveland, Ohio USA; 3 Center for Childhood Cancer and Blood Disorders, The Research Institute, Nationwide Children’s Hospital, Columbus, Ohio USA; 4 Department of Medicine, University Hospitals Cleveland Medical Center, Cleveland, Ohio USA; 5 Department of Pathology, University Hospitals Cleveland Medical Center, Cleveland, Ohio USA

**Keywords:** Cancer immunotherapy, Immunotherapy

## Abstract

NK cell adoptive therapy is a promising cancer therapeutic approach, but there are significant challenges that limiting its feasibility and clinical efficacy. One difficulty is the paucity of clinical grade manufacturing platforms to support the large scale expansion of highly active NK cells. We created an NK cell feeder cell line termed ‘NKF’ through overexpressing membrane bound IL-21 that is capable of inducing robust and sustained proliferation (>10,000-fold expansion at 5 weeks) of highly cytotoxic NK cells. The expanded NK cells exhibit increased cytotoxic function against a panel of blood cancer and solid tumor cells as compared to IL-2-activated non-expanded NK cells. The NKF-expanded NK cells also demonstrate efficacy in mouse models of human sarcoma and T cell leukemia. Mechanistic studies revealed that membrane-bound IL-21 leads to an activation of a STAT3/c-Myc pathway and increased NK cell metabolism with a shift towards aerobic glycolysis. The NKF feeder cell line is a promising new platform that enables the large scale proliferation of highly active NK cells in support of large scale third party NK cell clinical studies that have been recently intiatied. These results also provide mechanistic insights into how membrane-bound IL-21 regulates NK cell expansion.

## Introduction

Natural killer (NK) cells, comprising 10–15% of peripheral blood lymphocytes, play an important role in immune surveillance due to their innate ability to kill cancer and virally infected cells without prior sensitization^1^. NK cells are identified by the surface expression of CD56 and absence of the T cell marker CD3. A subset of NK cells express the FcγRIII protein, CD16, that enhances NK cell cytotoxic function by aiding in antibody-dependent cellular cytotoxicity (ADCC). NK cell function is largely controlled by families of cell surface activating and inhibitory receptors. Activation signals are promoted by activating receptors such as NKG2D that recognize ligands including the stress-induced protein MICA. Inhibitory receptors recognize molecules, such as MHC class I, that are universally expressed on normal cells and frequently downregulated on cancer cells^2^.

Due to their high cytotoxic activity against cancer cells, the adoptive transfer of NK cells is a promising therapeutic strategy^3,4^. In colorectal and pharyngeal cancer, the presence and activity of tumor-infiltrating NK cells is correlated with a better prognosis and reduction in metastatic risk^5–7^. Several types of cancers including hematologic malignancies, sarcomas, neuroblastoma, and ovarian cancer have been found to be particularly sensitive to NK cell therapy and there is at least some evidence of clinical success with adoptive NK cell therapy^8–10^. In addition to direct cancer therapy, NK cells have also been utilized to facilitate the engraftment of hematopoietic stem cell transplants, provide post-transplant cancer surveillance, and suppress viral infections^11^.

Despite being a promising strategy for combating cancer, the widespread clinical success of NK cell therapy has been limited partially by challenges in manufacturing large doses of NK cells that are likely necessary for clinical efficacy^12^. Generating enough primary NK cells for adoptive cell therapy (ACT) is a significant challenge because NK cells make up only 10–15% of lymphocytes in peripheral blood. Clinical trials suggest that high doses of NK cells (>10^9^/kg) are both safe and likely necessary for efficacy^13–16^. Another major advantage of manufacturing large numbers of NK cells is their potential use as a universal donor “off-the-shelf” therapy. This expansion method supports the use of NK cells from a single donor expansion to treat multiple patients. Universal donor NK cells are possible since unlike T cells, NK cells target cells in an MHC-independent manner and are not thought to cause graft-versus-host disease (GVHD).

Several *ex vivo* expansion platforms have been previously described, though very few clinical grade expansion platforms exist that can support large scale expansion of highly cytotoxic NK cells. For example, NK cells have been expanded with IL-2 as well as various other cytokine combinations such as IL-12, IL-15, IL-18, and IL-21. These cytokine-based expansion methods result in highly cytotoxic NK cells with memory-like features, but limited fold expansions (~4-fold at day 10 of expansion) have been reported due to NK cell senescence^17–19^. Expansion methods using irradiated accessory cells as antigen-presenting “feeder” cells lead to more robust yields^20–22^. For example, expanding NK cells with irradiated PBMCs and OKT3 can expand NK cells 2300-fold by day 17^23^. Another system involves Epstein-Barr virus-transformed lymphoblastoid feeder cells which result in robust expansion for 2–4 weeks before the NK cells become senescent^24^. To combat the issue of senescence, K562 feeder cells were engineered to express membrane-bound IL-21 (mbIL-21) with 4-1BB ligand allowing longer culture of NK cells.^21,22,25–27^. While these feeder cells have been used to support clinical trials, the use of these feeder cells for future clinical trials is restricted to a single institution.

Other approaches to expand NK cells for ACT involve the use of immortalized NK cell lines such as NK-92 cells. One major challenge with this approach is that the cells must be irradiated prior to patient administration which limits the efficacy of this therapeutic strategy because the cells cannot expand in patients and sustain anti-tumor activity^28,29^.

Here we report the creation of a novel mbIL-21 based NK cell feeder cell line that can support the generation of large doses of highly activated NK cells. We have recently utilized this platform to manufacture “universal donor” NK cells for a recently initiated phase 1 clinical trial. In addition, we characterize mechanisms through which mbIL-21 drive NK cell growth and activation by activating IL-21-dependent signaling leading to changes in metabolism enabling the cells proliferate and kill cancer cells.

## Materials and Methods

### Cell lines

OCI-AML3 cells were obtained from DSMZ and HL-60, 293T, HCT116, HT-29, and MDA-MB-468 cells were from ATCC. TC106 cell line was previously described in^30^. K562 cells were from MD Anderson. All cells were cultured in RPMI 1640 media (Hyclone) supplemented with fetal calf serum (Hyclone), penicillin (100 U/mL), streptomycin (100 ug/mL). Mycoplasma testing was performed on all cell lines at regular intervals using the Mycoplasma Detection Kit-Quick Test by bimake.com.

### NK cell isolation/purification

Peripheral blood mononuclear cells (PBMC’s) were isolated from the peripheral blood of healthy donors via ficoll (GE Healthcare) gradient centrifugation. NK cells were isolated from PBMC’s through magnetic bead CD3 depletion followed by CD56 isolation (Miltenyi biotec). NK cells were cultured with IL-2 for 24 hr (IL-2-NK) or with irradiated NKF cells and IL-2 (NKF-NK) as specified. All studies with NKF-expanded NK cells were performed after 2 weeks of expansion unless otherwise indicated.

### Cytotoxicity assay

NK cell cytotoxic function was assessed by the measuring the number of live cells identified by calcein-AM (CAM) labeling. Target cells and NK cells were labelled with CAM (BD Pharmingen) and calcein-violet (CV) (eBioscience), respectively. NK cells were co-cultured with target cells at the indicated ratios for 4 hours in triplicate, and the samples were analyzed by flow cytometry (Attune NXT, Invitrogen) in 96 well plates. The CV-positive NK cells were gated out for analysis. Percent cell lysis was calculated as follows:

$$\frac{(\#of\,CAM\,bright\,Target\,cells\,alone)-(\#\,of\,CAM\,bright\,cells\,in\,NK-Target\,co-culture)}{\#\,of\,CAM\,bright\,Target\,cells\,alone}\,\times \,100$$

### Phenotyping assay

The following antibodies were used: Biolegend (NKG2D-APC/Cy7, NKp46-FITC, NKp30-PE, CD158-FITC, CXCR6-PE, CD54-FITC), Novus Biologics (c-myc- BB421), BD Biosciences (NKP44-BB515, CD57-BV421, DNAM-1-PE, 2B4-BV421, Ki67- BB786, p-STAT3-Percp-cy5.5) and R&D systems (NKG2C-PE, NKG2A-alexa-488).

Intracellular staining for the phenotyping assay was done using the “Transcription Factor Phospho Buffer Set” (BD Biosciences). Instructions were followed according to protocol specified by the manufacturer. For all stains, 50–200 μL of cells at a concentration of 2.5 × 10^6^ cells/mL were resuspended in a 96 well V-bottom plate. First, cell surface makers were stained at optimized concentrations of fluorescent antibodies in PBS (3% FBS) at room temperature in the dark, for 20–30 min. Second, cells were fixed using 100 μL fix/perm solution for 40 min at 4 °C. Third, pre-cooled (−20 °C) Perm III buffer was added to the cells and cells were incubated at 4 °C for 15 min. Finally, the cells were stained with intracellular antibodies in perm/wash buffer at 4 °C for 40 min. 2 washes with perm/wash buffer was performed after each step. The cells were resuspended in 3% FBS/PBS at the end of the stain and data was acquired using the BD Fortessa. The analyses were done using FlowJo.

### Mouse models

Nod-SCID-IL-2Rgamma-null mice (NSG, Jackson Laboratory) were injected with 1 × 10^5^ TC106 (sarcoma) cells subcutaneously bilaterally. Ten days following TC106 injection, when tumors were palpable, the mice (n = 5 per group) received 1 × 10^6^ NK cells injected intravenously (IV) once a week or vehicle as well as IL-2 (75,000 U/ml). Tumor volumes were measured twice a week. Mice were sacrificed after they lost 15% of their initial weight.

The leukemia xenograft model was established by injecting 1 × 10^6^ Jurkat cells IV into NSG mice. Seven days following injection, mice (n = 5 per group) received 5 × 10^6^ NK cells or vehicle weekly as well as IL-2 (75,000 U/ml). Mice were sacrificed after becoming moribund or losing 15% of their initial weight in accordance with out institutional guidelines.

### Immunostaining of harvested mouse tissues

The harvested lung tissues were fixed in paraformaldehyde prior to sectioning onto glass slides. H&E slides were baked at 60 °C for 90 mins. Glass slides were de-paraffinized in xylene for 30 mins and rehydrated in ethanol for 6 mins. Slides were rinsed with water and placed in Harris Modified Hematoxylin (Fisher) for 15 mins. Slides were rinsed with water and thereafter dipped in 1% solution of 70% ethanol and hydrochloric acid (Fisher) 8 times. Slides were dipped in 1% lithium carbonate (Fisher) 5 times and rinsed with water. Slides were placed in ethanol followed by 80% ethanol, glacial acetic acid solution for 3 mins. Slides were placed in 100% ethanol for 10 mins followed by xylene for 4 mins. Coverslips were added and glass slides were allowed to dry overnight.

Ki67 slides were rinsed in water following de-paraffinization and rehydration with ethanol. Antigen unmasking solution (Vector Laboratories) was applied to the slides for 30 secs. Slides were rinsed with water and blocked with rodent block M (BioCare Medical) for 30 mins and rinsed in TBST. Slides were incubated in diluted rat Ki67 primary antibody (eBioscience) for 1 hour and rinsed in TBST. Rat probe (Biocare Medical) was applied to glass slides for 15 mins and rinsed with TBST. HRP was applied for 15 mins and rinsed with TBST. DAB chromogen (Biocare Medical) was added to the glass slides and inubated in the dark for 5 mins, prior to washing with water. Slides were counterstained with CAT hematoxylin (Biocare Medical) for 1 min, rinsed with water followed by TBST.

### Metabolism studies

Metabolic studies were performed using the XFe96 Analyzer (Seahorse Bioscience). NK cells were cultured in 200 U/mL IL-2 overnight (IL-2-NK), expanded with NKF feeder cells (NKF-NK), or expanded with OCI-AML3 feeder cells (OCI-NK). Cell energy tests were performed in Seahorse XF Base Medium in Minimal DMEM (Agilent) supplemented with 1 mM pyruvate, 2 mM glutamine, and 10 mM glucose. Drug concentrations during the assay were 0.25 µM FCCP (to depolarize mitochondrial membrane) and 1 µM oligomycin (to inhibit ATP synthase). Cells were plated in at least duplicates and the oxygen consumption rate (OCR) and extracellular acidification rate (ECAR) were measured. Wave software and report generator files, provided by the manufacturer, were used to analyze the data.

### Statistical analysis

Nonparametric 2-tailed unpaired t tests were performed to determine significance. The following denotations for significance levels were used: *p < 0.05, **p < 0.01 and, ***p < 0.001. 2-way Anova was calculated, to determine significance among groups. The log-rank (Mantel-Cox) test was performed to determine significance of mouse xenograft survival data. All data represent at least 3 independent experiments, unless otherwise specified.

### Regulatory approvals

The work using human primary NK cells was performed according to the human subjects guidelines at University Hospitals Cleveland Medical Center and approved by their Institutional Review Board. Informed consent was obtained from all subjects. All animal experiments were approved by the Case Western Reserve University Institutional Animal Care and Use Committee and performed according to their guidelines.

## Results

### Development and optimization of a mbIL-21 based feeder expansion platform

In order to develop a novel NK cell expansion platform, we aimed to select a suspension cell line to use as a feeder cell that was efficiently lysed by NK cells and exhibits a low level of expression of HLA class I expression. A low level of HLA class I proteins is beneficial as certain epitopes are recognized by NK cell inhibitory killer inhibitory receptors (KIRs) which impair NK cell activation. Initially, the leukemia cell lines HL-60 and OCI-AML3 were selected since they meet these desired characteristics (Supplemental Fig. 1A). The two cell lines were irradiated and cultured with freshly isolated NK cells for 1 week to assess their expansion potential. OCI-AML3, a myeloid leukemia cell line, led to a 4.6-fold expansion compared to 2.9-fold expansion with the HL-60 cells (Supplemental Fig. 1B).

**Figure 1:**
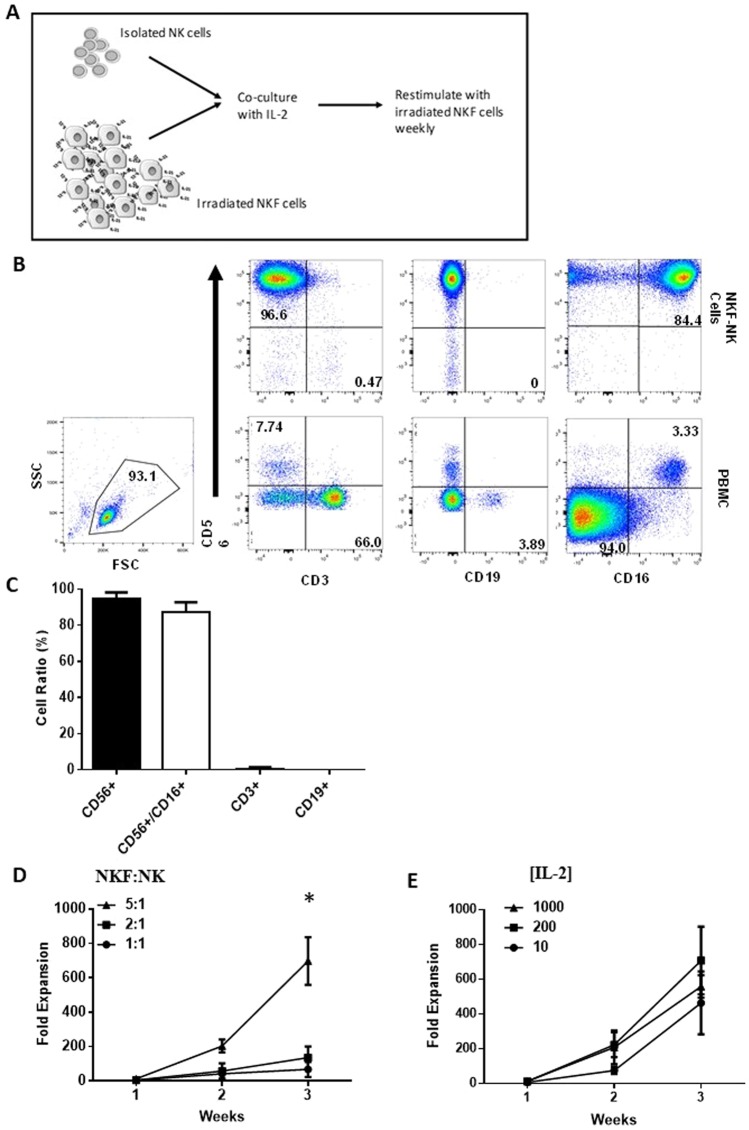
NKF feeder cells enable NK cell proliferation. (**A**) Schema of the NKF based NK cell expansion platform. (**B**) NKF-expanded NK cells are highly pure. Representative flow plots depicting purity of NK cells in the initial PBMC population and after 2 weeks of expansion. (**C**) Relative fractions of cell types in 2 week expanded NKF-expanded product, n = 15. (**D**) Fold expansion of NK cells at the indicated NKF:NK ratios and 200U/ml IL-2 after 3 weeks, n = 4. (**E**) Fold expansion of NK cells at the indicated IL-2 concentrations and NKF cells at a 5:1 ratio after 3 weeks:NK, n = 5. Data represents mean +/− SEM. *p < 0.05.

It has been reported that the presence of membrane-bound IL-21 (mbIL-21) can prevent NK cells from undergoing senescence, markedly improving their ability to expand *ex vivo*^25^. A novel NK feeder cell was developed using OCI-AML3 cells transduced with mbIL-21 (NKF) (Supplemental Fig. 1C). To expand peripheral blood isolated NK cells, NK cells (9% of the total PBMC (CI 95%: 6.178–15.14)) were isolated from PBMCs and co-cultured with irradiated NKF cells which were added weekly (Fig. 1A). The purity of expanded NK cells for 15 donors was assessed by flow cytometry. After 2 weeks of expansion, CD56+/CD3− cells were approximately 94% (CI 95%: 92.32–96.53) of the expanded cells, CD3+ cells made up <1% (CI 95%: 0.069–1.03), and B cells were virtually undetectable (Fig. 1B,C). Approximately 87% of expanded NK cells were CD56+/CD16+ suggesting a large portion of these cells could mediate ADCC. The low T cell contamination post-expansion is important for avoiding potential graft-versus-host-disease for universal donor NK cells.

The NKF expansion platform was optimized based on NKF-to-NK ratios and IL-2 concentrations. The goal for feeder cell addition was to generate robust expansion while limiting the feeder cell numbers to avoid the unnecessary presence of excess dead feeder cells in the final product. After 3 weeks, expansions at a 5-1 (NKF-to-NK) ratio resulted in an 8.3-fold higher NK cell yield compared to a 1-1 ratio (p = 0.017) and a 2.7-fold higher NK cell yield compared to a 2-1 ratio (p = 0.057) (Fig. 1D). Expansion yield at IL-2 concentrations ranging from 10–1000 U/mL did not result in a statistically significant difference (Fig. 1E). This is consistent with previous reports that high IL-2 concentrations do not impact the proliferative capacity of feeder cell expansion of NK cells^31–34^. The yield at 200 U/mL IL-2 was the highest, therefore subsequent expansions were performed with a 5-1 feeder-to-NK cell ratio and 200 U/mL IL-2.

### NKF-NK cells exhibit potent cytotoxic activity against both hematologic and solid cancer cells

**Figure 2:**
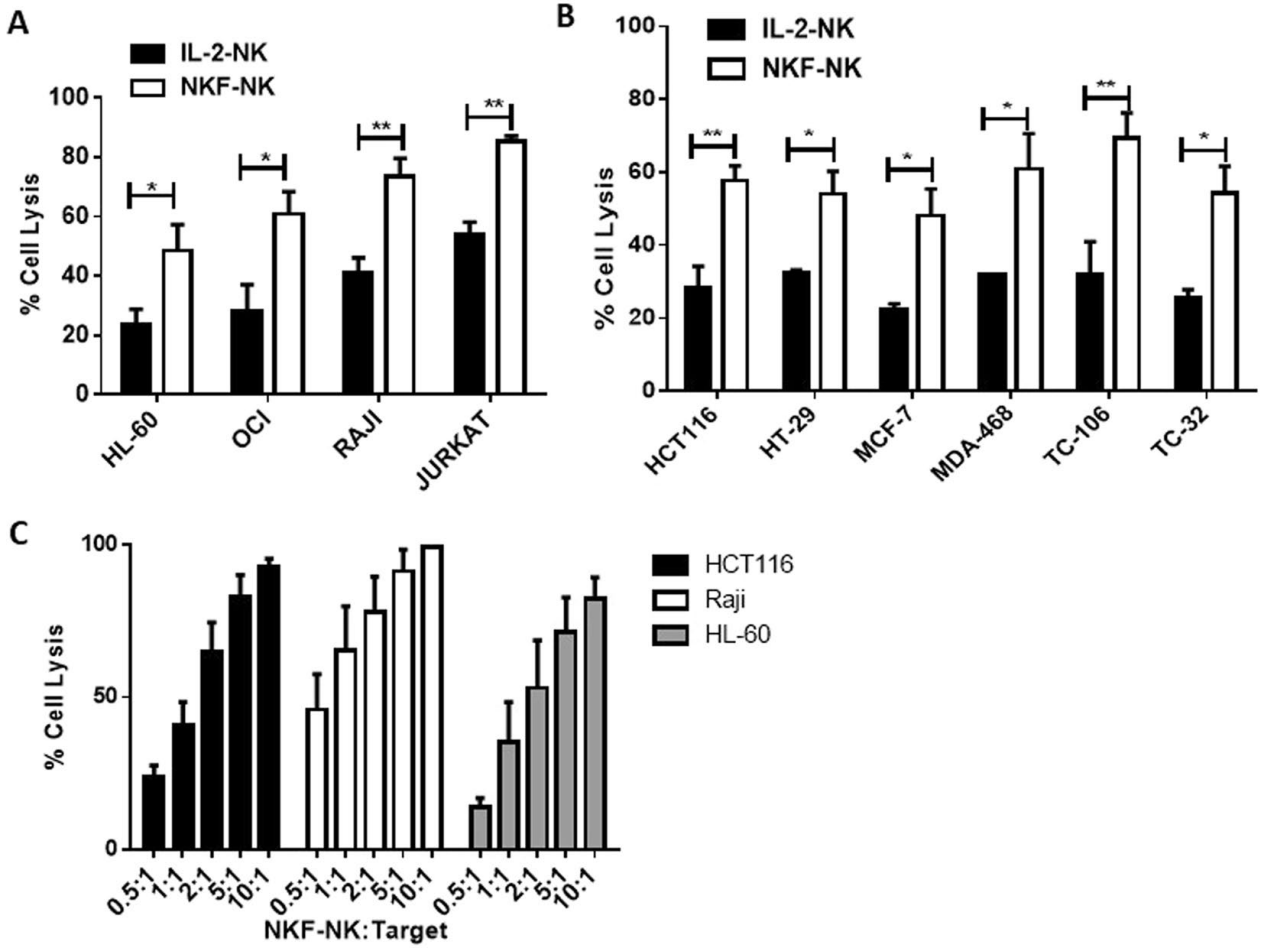
NKF-NK cells exhibit potent cytotoxic activity against both hematologic and solid cancer cells. The cytotoxic activity of 2 week expanded NKF-NK or IL-2-NK cells was assessed against hematologic malignancy cell lines (**A**) or solid tumor cell lines (**B**) after 4 hr of co-culture at a 1:1 NK:Target cell ratio, n = 4. (**C**) NKF-NK cells demonstrate dose-dependent cytotoxicity against cancer cells. The cytotoxic activity of NKF-NK cells was measured using the indicated NK:Target ratios, n = 4. Data represents mean +/− SEM. *p < 0.05, **p < 0.01.

Besides being able to generate large numbers of NK cells, another major challenge for adoptive NK cell therapy is insufficient cytotoxic activity against cancer cells. Therefore, the cytotoxic activity of 2 week expanded NKF-expanded NK cells (NKF-NK) was assessed in comparison to the traditional source of NK cells used for adoptive cell therapy, IL-2 overnight activated NK (IL-2-NK) cells using a flow cytometry based cytotoxicity assay (see methods). NKF-NK cells exhibited markedly increased cytotoxic activity against a wide variety of cancer cell lines as compared to IL-2-NK cells (Fig. 2A,B and Supplemental Fig. 3). NKF-NK cells achieved 31% (p = 0.003) and 37% (p = 0.009) more killing of Jurkat and TC106 cells respectively than IL-2-NK cells at a NK-target ratio of 1-1. To further assess NKF-NK cytotoxic activity, these cells were co-cultured with leukemia, lymphoma and colon cancer cell lines at a variety of NK-target cell ratios. NKF-NK cells demonstrated dose-dependent killing of all cell types tested (Fig. 2C). We further compared the cytotoxic activity of NKF expanded NK cells to mbIL21-K562 expanded NK cells. As seen in Supplemental Fig. 2, NKF feeder cells lead to very similar NK cytotoxic activity (as well as NK cell expansion rate) as mbIL21-K562 feeder cells.

### NK expansion with NKF cells leads to marked changes in cell surface phenotype

**Figure 3:**
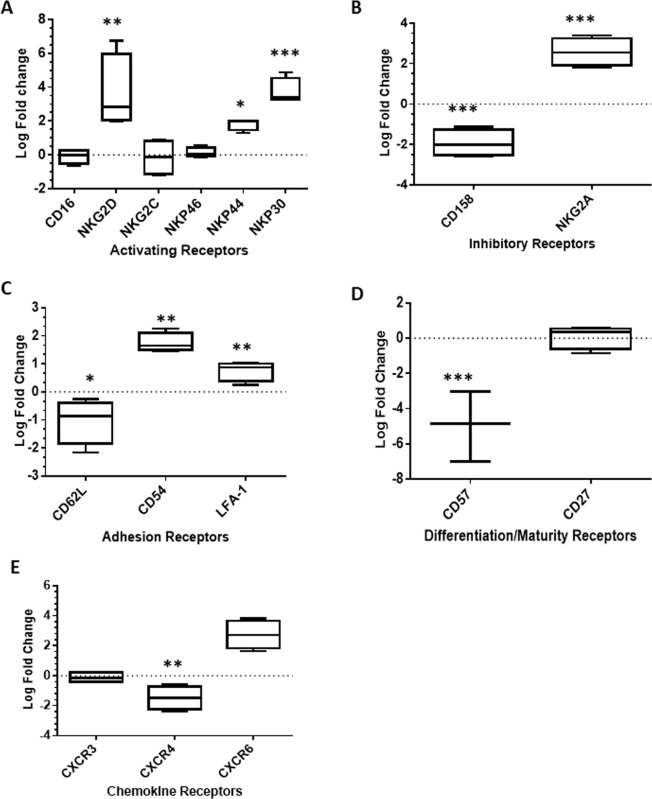
mbIL-21 signaling in NKF-NK cells leads to marked changes in cell surface phenotype. Expression of NK activating receptors (**A**), inhibitory receptors (**B**), adhesion receptors (**C**), NK terminal differentiation receptors (**D**), and trafficking receptors (**E**) on IL-2-NK and 2 week expanded NKF-NK cells were measured by flow cytometry, n = 4. Horizontal dotted lines on the plots represent no fold change in the expression of the surface receptor. Data represents mean +/− SEM. *p < 0.05, **p < 0.01, ***p < 0.001.

NK cell activation and function is coordinated by the engagement of cell surface molecules such as activating and inhibitory receptors. Using flow cytometry, the baseline expression levels of key NK cell surface molecules were measured in both IL-2-NK and 2 week expanded NKF-NK. In order to make direct comparisons, the same donors were utilized for both populations of NK cells (n = 4). Expansion with NKF cells led to increased expression of the activating receptors NKG2D, NKp30, and NKp44 as compared to IL-2NK cells (Fig. 3A and Supplemental Fig. 4). Activating receptors such as NKG2D and the natural cytotoxicity receptors are crucial to NK cell activation and function^35^. Ligands for NKG2D are expressed in a variety of cancers such as sarcomas, lymphomas, leukemia, melanoma, hepatoma, and prostate cancer^36^. NKp46 plays a role in preventing metastases and NKp44 promotes lysis of a broad spectrum of cancer cell lines and cytokine release^37,38^. There was also an increase in the inhibitory receptor, NKG2A, a marker that is reduced in terminally differentiated NK cells, as reported by other groups^39,40^. NKF-NK cells also had a significant decrease in the expression of killer immunoglobulin receptors (KIRs) (Fig. 3B). Expansion with NKF cells also led to an increase in adhesion receptors (LFA-1 and CD54) which are important for NK cell conjugation with tumor targets, enhancing cytolysis (Fig. 3C and Supplemental Fig. 4) CD57 expression, denoting terminal differentiation of NK cells, was also measured and found to be decreased in NKF-NK cells (Fig. 3D). NK cells expressing high levels of CD57 are limited in their continued proliferative capacity *in vivo*^41^. Finally, the expression of key chemokine receptors that regulate the *in vivo* trafficking of NK cells were analyzed. NKF expansion led to a decrease in CXCR4, a receptor reported to sequester NK cells in the bone marrow^42^ (Fig. 3E). In addition, an increase in CXCR6 suggests that NKF-NK cells can traffic to the liver, a common site of cancer metastases. CXCR6 has also been indicated in the development of memory-like NK cells that persist following hapten or viral exposure^43^.

### mbIL-21 signaling promotes sustained NK cell expansion and increased metabolic activity

While short term co-culture with NKF cells leads to NK cell proliferation and activation, to support “universal donor” NK cell clinical studies sustained proliferation is necessary. Unlike IL-2 treated NK cells that rapidly undergo senescence, NKF cells enable long term NK cell proliferation. For example, after 5 weeks of expansion there was an average of 10,973-fold expansion (Supplemental Fig. 3A).

In order to support the clinical translation of NKF-expanded NK cells, it is also necessary to utilize high capacity culture devices to accommodate the high cell numbers. G-REX culture systems allow the culture of high densities of cells such as T cells due to the presence of a gas permeable membrane surface^44^. Therefore, we tested the ability of freshly isolated NK cells to expand in the G-REX device. NK cells were expanded for 2 weeks with NKF cells and the average NK cell yield was 89-fold (Supplemental Fig. 3B). This demonstrates that the semi-automated G-REX system is capable of expanding NK cells utilizing NKF feeder cells.

**Figure 4:**
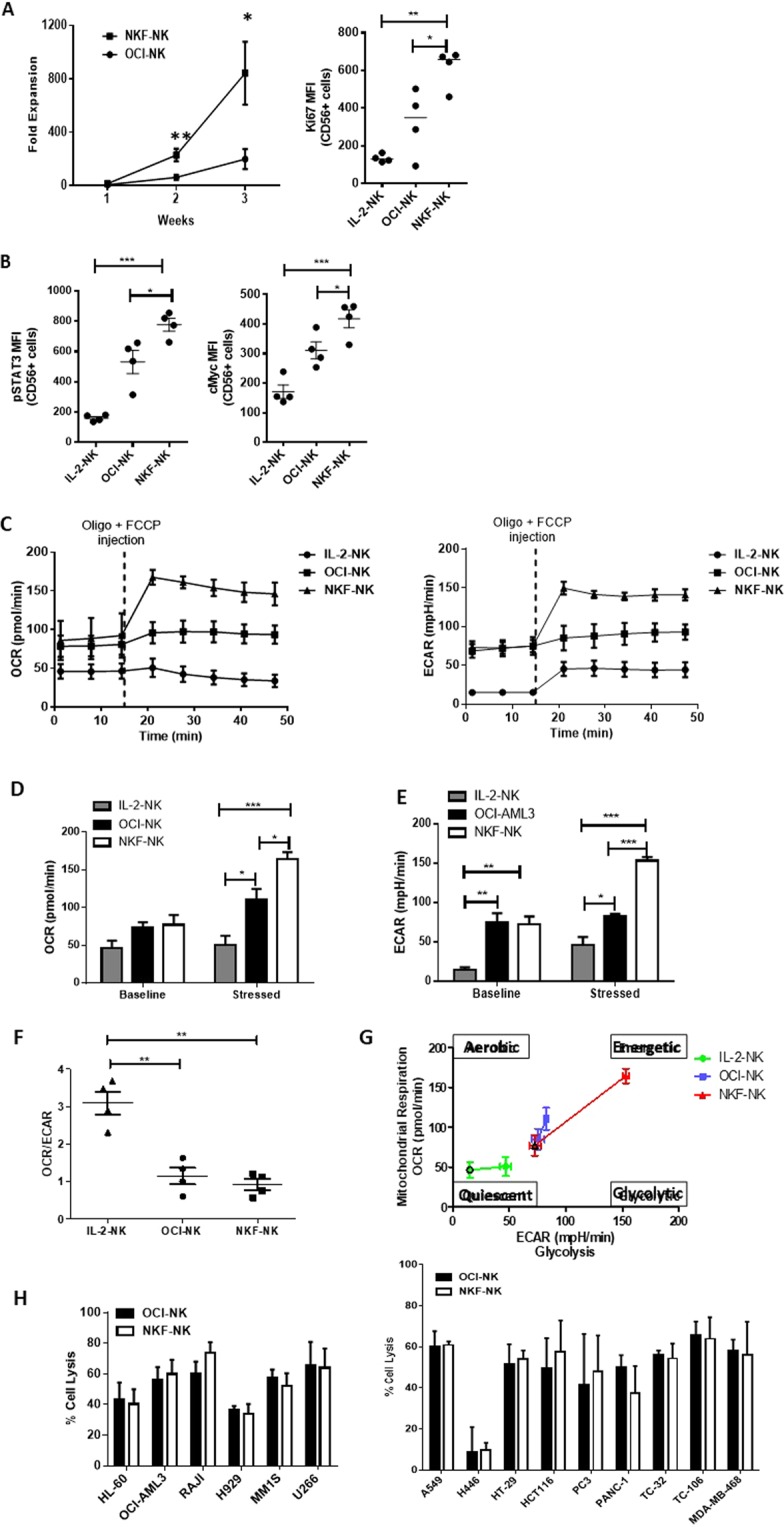
mbIL-21 signaling promotes increased metabolic activity in NKF-NK cells but does not affect cytotoxicity. (**A**) NKF cells lead to enhanced NK cell proliferation as compared to OCI-AML3 cells at a 5:1 feeder-to-NK ratio as measured by cell counts after 3 weeks of expansion. The proliferation marker, Ki67, expression is higher in NKF-NK cells as compared to IL-2-NK and OCI-NK cells as assessed by flow cytometry, n = 4. (**B**) NKF-NK cells exhibit increased pSTAT3 and c-myc levels compared to IL-2-NK and OCI-NK as measured by flow cytometry, n = 4. (**C**) OCR and ECAR measurements of IL-2-NK, OCI-NK, and NKF-NK cells at baseline and after the addition of oligo (1 µM) and FCCP (0.25 µM). Average OCR (**D**) and ECAR (**E**) measurements of IL-2-NK, OCI-NK, NKF-NK cells at baseline and stressed conditions. (**F**) OCR/ECAR ratio of IL-2-NK, OCI-NK and NKF-NK cells. (**G**) ECAR vs OCR plot illustrating the energetic state of the indicated NK cells, n = 4. (**H**) NKF-NK cells demonstrate similar cytotoxic activity to OCI-NK cells as were measured by the 4-hr cytotoxicity assay using hematologic malignancy or solid tumor cell lines at a 1:1 NK:Target cell ratio, n = 4. NK cells were expanded 2–3 weeks for these experiments. Data represents mean +/− SEM. *p < 0.05, **p < 0.01, ***p < 0.001.

The proliferation of NK cells expanded with NKF cells was compared to that from parental OCI-AML3 cells (OCI-NK). NKF cells resulted in an average of 843-fold NK cell expansion after 3 weeks as compared to 200-fold using OCI-AML3 cells (p = 0.027) (Fig. 4A). Consistent with enhanced NK cell proliferation, the proliferation marker Ki67 was upregulated on NKF-NK cells as compared to OCI-NK cells (p = 0.038). As expected, Ki67 levels are higher when either feeder cell line is used as compared to IL-2-NK cells (p = 0.0021) (Fig. 4A).

Next activated molecules downstream of IL-21 Receptor (IL-21R) signaling were assessed to elucidate mechanisms of IL-21-dependent sustained NK cell proliferation. IL-21R signaling is known to activate STAT3 which in turn can directly induce c-myc expression. NKF-NK cells exhibited higher p-Stat3 expression than OCI-NK cells (p = 0.041) and IL-2-NK cells (p = 0.00038) (Fig. 4B). In addition, NKF-NK cells demonstrated higher expression of c-myc as compared to OCI-NK cells (p = 0.043) and IL-2-NK cells (p = 0.00085) (Fig. 4B).

As the activity and proliferative status of immune cells is largely governed by their metabolic capacity, the impact of mbIL-21 signaling on NK cell metabolism was investigated. After activation, the metabolism of immune cells typically shifts from generating energy mainly through oxidative phosphorylation to aerobic glycolysis. The oxygen consumption rate (OCR) which measures oxidative phosphorylation (oxphos) rate, and the extracellular acidification rate (ECAR) which measures glycolysis were measured in NKF-NK, OCI-NK and IL-2-NK cells (Fig. 4C). At baseline, NKF-NK cells and OCI-NK cells had the same oxphos and glycolysis rates (Fig. 4D,E). Under stress, the NKF-NK cells had higher OCR (p = 0.043) and ECAR (p = 0.0015) values compared to OCI-NK cells (Fig. 4D,E). Both expanded NK cell populations had higher OCR and ECAR values than IL-2-NK (Fig. 4D,E). The OCR/ECAR ratio of NKF-NK cells was close to 1, suggesting a balance of the glycolytic and oxphos pathways (Fig. 4F). The energetic state of NKF-NK is higher than that of OCI-NK and IL-2-NK as observed on a OCR vs ECAR plot (Fig. 4G).

As mbIL-21 leads to enhanced NK cell proliferation and metabolism during feeder cell expansion, we also assessed its impact on NK cell cytotoxic activity. While NKF-NK cells demonstrate a marked increase in cytotoxic activity as compared to non-feeder expanded cells (Fig. 2A,B), there was no significant difference in cytotoxic function of NKF-NK and OCI-NK cells (Fig. 4H). This result suggests that mbIL-21 may not significantly affect the cytotoxicity of NK cells at least in the presence of feeder cells.

### NKF-NK cells reduce tumor burden in mouse tumor xenografts and improve survival

**Figure 5:**
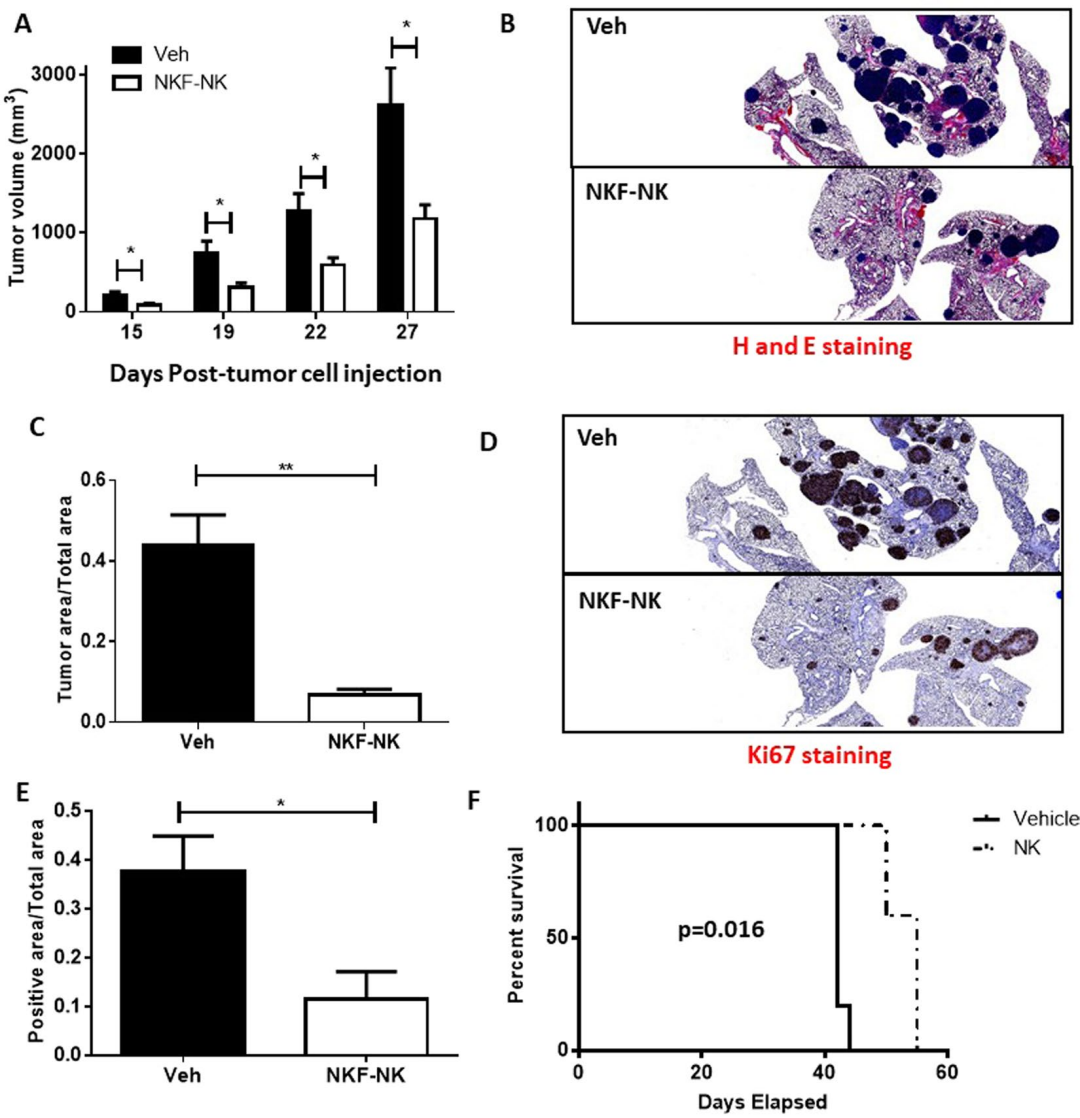
NKF-NK cells reduce tumor burden in mouse xenografts and improve mouse survival. (**A**) NKF-NK cells exhibit efficacy in a mouse sarcoma model. TC106 cells were injected subcutaneously into NSG mice. Mice were either treated weekly with vehicle (Veh) or NKF-NK cells (NK). Tumor volumes were measured on indicated days (primary tumor). 5 mice per treatment group. (**B**) H&E staining of resected lung tissue from Veh and NKF-NK mice at 1X magnification (metastasis). (**C**) Quantification of tumor in resected lung tissue based on H&E staining (metastasis). (**D**) Ki67 staining of resected lung tissue from Veh and NKF-NK mice at 1X magnification (metastasis). (**E**) Quantification of Ki67 staining in resected lung tissue (metastasis). (**F**) NKF-NK demonstrate efficacy in a mouse model of leukemia. Jurkat cells were injected intravenously into NSG mice. Mice were either treated weekly with vehicle (Veh) or NKF-NK cells (NK). Survival of the mice was determined. NK cells were expanded 2–5 weeks for these experiments. Data represents mean +/− SEM. *p < 0.05, **p < 0.01.

To assess the therapeutic potential of NKF-NK cells for cancer therapy, mouse models of sarcoma and lymphoid leukemia were used. For the sarcoma model, the Ewing’s sarcoma cell line, TC106, was injected subcutaneously into immunodeficient NSG mice. The sarcoma model was employed because it leads to metastases to the lungs, the most common site of sarcoma metastasis in humans and a known site for NK cell trafficking *in vivo*^45,46^. In this model, not only did we observe a reduction in the growth of the primary sarcoma tumor with NKF-NK cell administration, but there was a dramatic reduction in tumor metastases to the lung (Fig. 5A–C). Ki67 staining of lung specimens from vehicle-treated mice and NKF-NK-treated mice revealed decreases in proliferation of tumor cells in NKF-NK-treated mice as compared to vehicle-treated mice (p = 0.023) (Fig. 5D,E).

In addition to evaluating the NKF-expanded NK cells for their ability to reduce tumor growth, these cells were also evaluated for their ability to prolong survival in a highly aggressive circulating lymphoid leukemia model. NSG mice were injected intravenously with Jurkat cells to establish circulating disease. Mice injected with NKF-NK cells demonstrated an approximately 13-day median increase in survival over control treated mice (p = 0.0016) (Fig. 5F).

## Discussion

Adoptive NK cell therapy exhibits promise in the area of cancer therapy, but the development of additional robust methods to expand large numbers of highly activated NK cells is important to continue to advance the field. In addition, further understanding of the mechanisms that enable this expansion is important for the development of optimal strategies. Here we report the development of a novel feeder line, NKF, that can support “universal donor” NK cell therapy clinical trials and elucidate how mbIL-21 impacts NK cell expansion and activation. Due to the limited availability of robust *ex vivo* expansion platforms that are available for clinical use, the development of new feeder lines is important. Compared to the K562-mbIL21 feeder cells, NKF cells behaved similarly with regards to NK cell expansion and cytotoxicity against cancer cells. As the K562-mbIL21 feeder cells are no longer available for wide spread clinical use, the NKF platform may provide a valuable alternative.

NKF cells were found to enable both large-scale *ex vivo* growth as well as activation of freshly isolated NK cells starting with T cell depleted blood samples. The use of T cell depleted starting material is important as T cells also expand under these conditions and would contaminate the NK cell product. The expanded NK cells exhibit significant cytotoxic activity against a wide variety of hematologic and solid cancer cells *in vitro* as compared to non-expanded, IL-2 activated NK cells. These NK cells also exhibit anti-tumor activity in mouse models of human leukemia and sarcoma. The expanded cells exhibit phenotypic changes such as increased levels of NKG2D and NKp30, consistent with an activated state despite their continued ability to proliferate.

In order to utilize NKF cells to manufacture clinical grade NK cells, a master cell bank was created that is supporting a recently initiated clinical trial to test universal donor NKF-expanded NK cells in cancer patients (NCT02890758). While it is feasible to expand NK cells with feeder cells in traditional flasks or gas permeable G-REX flasks, further developments in NK cell manufacturing are warranted to support large scale, universal donor clinical studies. While the existing methods can support the manufacture of tens of billions of cells, higher capacity culture systems will be necessary to efficiently and cost effectively generate hundreds of billions or more NK cells from single donor expansions. Based upon an average of expansion of over 10,000 fold at 5 weeks, it should be feasible to manufacture greater than 4 × 10^12^ NK cells starting from a single donor apheresis sample. Methods to characterize the feasibility of feeder cell-based expansions in large capacity bioreactors such as the Xuri are ongoing.

As NK cells, unlike T cells, are not thought to elicit cause graft-versus-host disease, there are numerous benefits of developing “universal donor” off-the-shelf NK cells. Logistically, this strategy would dramatically lower the cost and increase the accessibility of this therapeutic strategy worldwide. Despite promising approaches for *ex vivo* expansion, the need of donors for each intended patient and the associated costs (both logistics and financial) that accrue with cell processing per patient still limit the feasibility of adoptive NK cell therapy. Robust expansion systems such as the NKF feeder cells should enable the ability to manufacture NK cell doses for 100 or more recipients from a single donor. Being able to harvest NK cells from one donor would greatly alleviate the costs of this therapeutic approach. Utilizing a donor not matched by HLA to recipients also increases the potential for NK cell alloreactivity to promote ‘graft versus leukemia (GVL) effects (without GVHD). This has been found to improve disease free survival in certain cancer patient populations^47–49^.

Despite the ability to generate large numbers of active NK cells, a major challenge in realizing high levels of clinical efficacy is the maintenance of NK cell activity and proliferation *in vivo*. Tumor microenvironments exhibit immunosuppressive effects through the production of cytokines such as TGFβ and IL-10^50^. In order to improve on current NK cell therapy, preclinical studies suggest the combination of NK cells with immunomodulatory agents such as TGFβ inhibitors offer promise. For example, the combination of expanded NK cells with Galunisertib leads to a marked increase in anti-tumor efficacy in a mouse model of colon cancer metastasis^51^. In addition, the immunomodulatory agent lenalidomide, enhances NK cell cytotoxicity against multiple myeloma cells^52^.

It has been previously demonstrated that feeder cell-based expansion with mbIL-21 leads to the ability of NK cells to avoid undergoing senescence as evidenced by a reduction in telomere shortening, though little is known about molecular mechanisms through which mbIL-21 supports continued NK cell proliferation^25^. Utilizing non-expanded NK cells and NK cells expanded with NKF or parental feeder cells lacking mbIL-21, we further assessed mechanisms through which mbIL-21 sustains NK cell expansion. Our studies demonstrate that mbIL-21 leads to the activation of a well characterized IL-21 dependent pathway consisting of STAT3 and cMyc. STAT3 activation is necessary for downstream effects of IL-21 signaling and is a known inducer of c-Myc. The activation of cMyc is known to regulate various cellular processes, important in NK cell proliferation and activity including the induction of glycolysis, mitochondrial biogenesis, and cell cycle^53–55^. Interestingly, NK expansion by the parental OCI-AML3 feeder cells that lack mbIL-21 led to partial activation of the STAT3/cMyc pathway likely due to the fact that the activation of many other receptor signaling pathways also can lead to minimal STAT3 activation.

Recently it has become well-recognized that metabolic reprogramming of immune cells is essential for their proliferative capacity and immune cell functions, most notably through the activity of c-Myc^53,56^. In particular, immune cells increase their metabolism, for robust proliferation by ensuring sufficient biosynthetic precursors. This metabolic shift also enables the immune cells to survive in hypoxic environments as often occurs in the tumor microenvironment. While most previous studies have focused on T cells, reports exist that this phenomenon occurs in NK cells^57^. For the first time we have shown that feeder cell-expanded NK cells exhibit a marked metabolic shift by increasing both glycolysis and oxidative phosphorylation as compared to both parental OCI feeder cells as well as non-expanded, IL-2 activated NK cells (IL-2-NK).

Overall, NK adoptive cell therapy is a promising therapeutic approach that depends on the development of robust *ex vivo* expansion platforms such as the NKF cells that can support the manufacture of highly active clinical grade NK cells.

## Supplementary information

Supplementary Figures
