# Supplementary material for: Membrane bound IL-21 based NK cell feeder cells drive robust expansion and metabolic activation of NK cells
Source: Sci Rep. 2019 Oct 17;9:14916. doi: 10.1038/s41598-019-51287-6 (PMC6797802; doi:10.1038/s41598-019-51287-6)
Supplement: Supplementary file 1 — Supplementary Figures [file 41598_2019_51287_MOESM1_ESM.pdf]

Membrane bound IL-21 based NK cell feeder cells drive robust expansion and metabolic activation of NK cells

Evelyn O. Ojo, Ashish Arunkumar Sharma, Ruifu Liu, Stephen Moreton, Mary-Ann Checkley-Luttge, Kalpana Gupta, Grace Lee, Dean A Lee, Folashade Otegbeye, Rafick-Pierre Sekaly, Marcos de Lima, and David N Wald

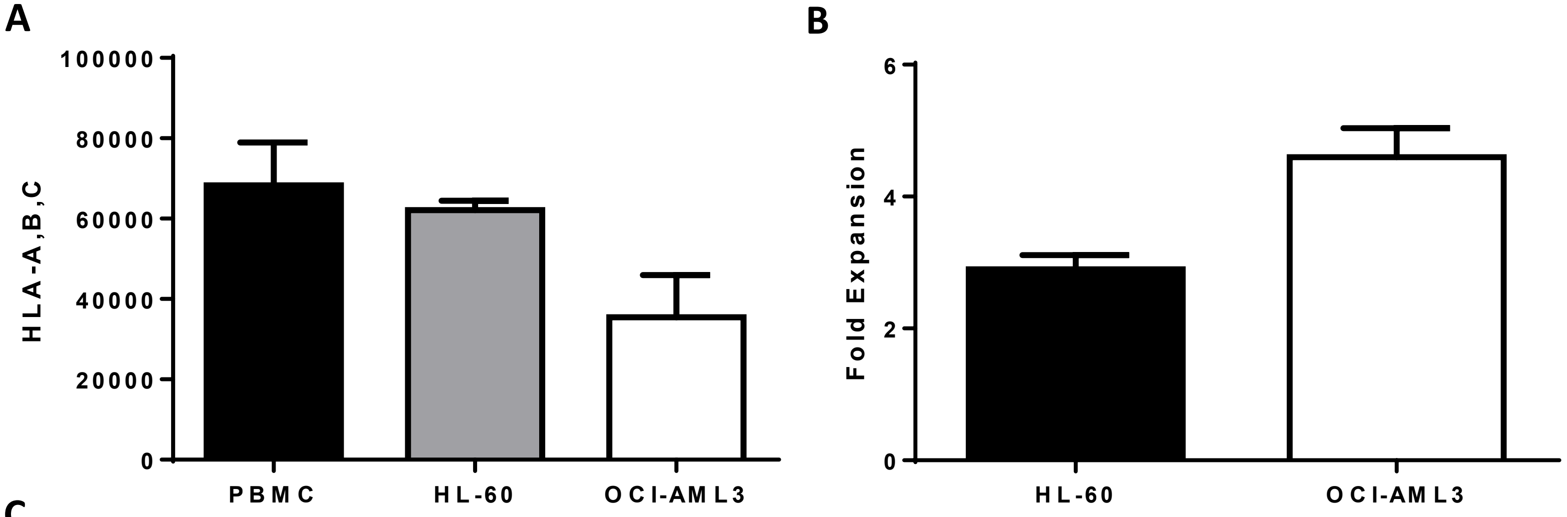

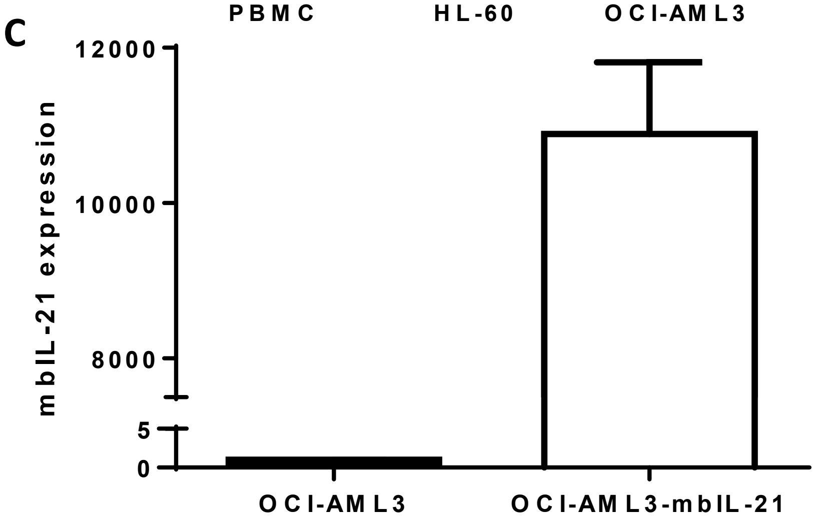

Supplemental Figure 1. A. HLA-A,B,C expression on PBMC, HL-60 and OCI-AML3 cells as assessed by flow cytometry. B. Fold expansion of NK cells using HL-60 and OCI-AML3 as feeder cells at a 5:1 ratio after 1 week co-culture. C. Rt-PCR assessment of mbIL-21 expression in OCI-AML3 and NKF-NK cells. Data represent mean +/- SEM.

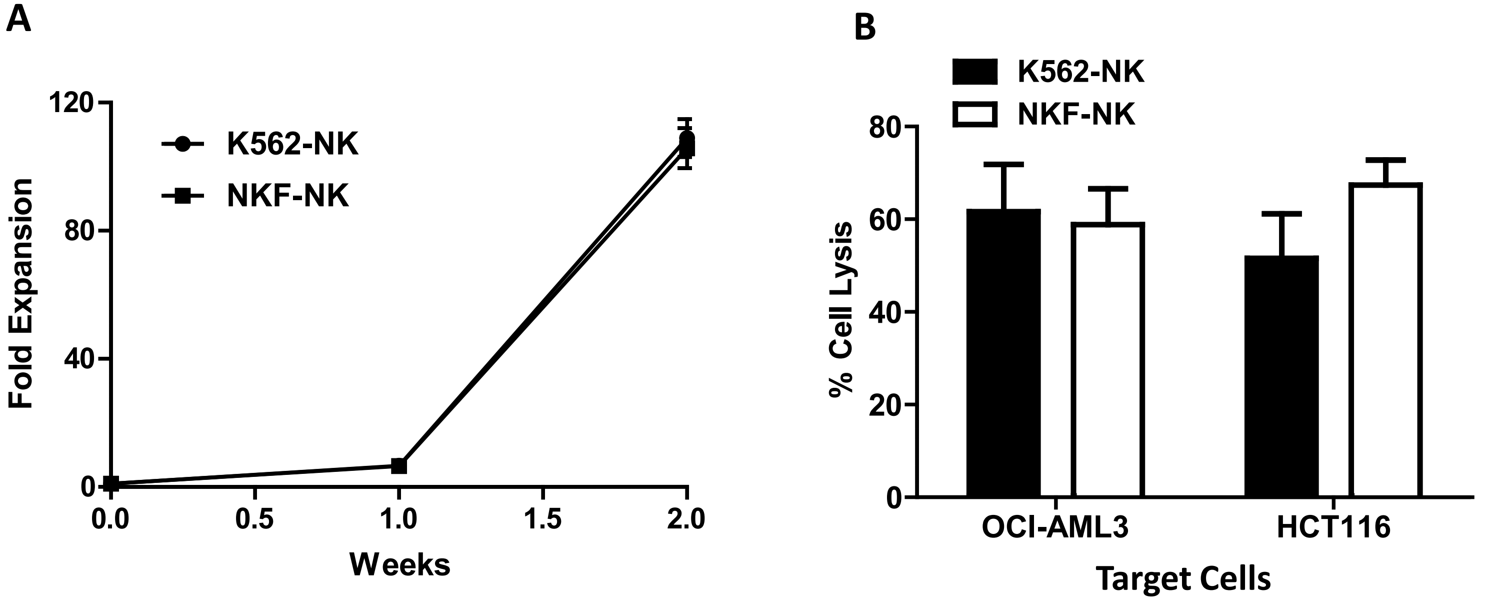

Supplemental Figure 2. A. Fold expansion of K562-NK and NKF-NK cells at 5:1 feeder-to-NK ratio and 100U/mL IL-2 after 2 weeks, n=1. B, The cytotoxic activity of 2 weeks-expanded NKF-NK and K562-NK was assessed against OCI-AML3 and HCT116 cells after 4hr co-culture, n=3. The NK cell-to-target ratio was 1-1. Data represent mean +/- SEM.

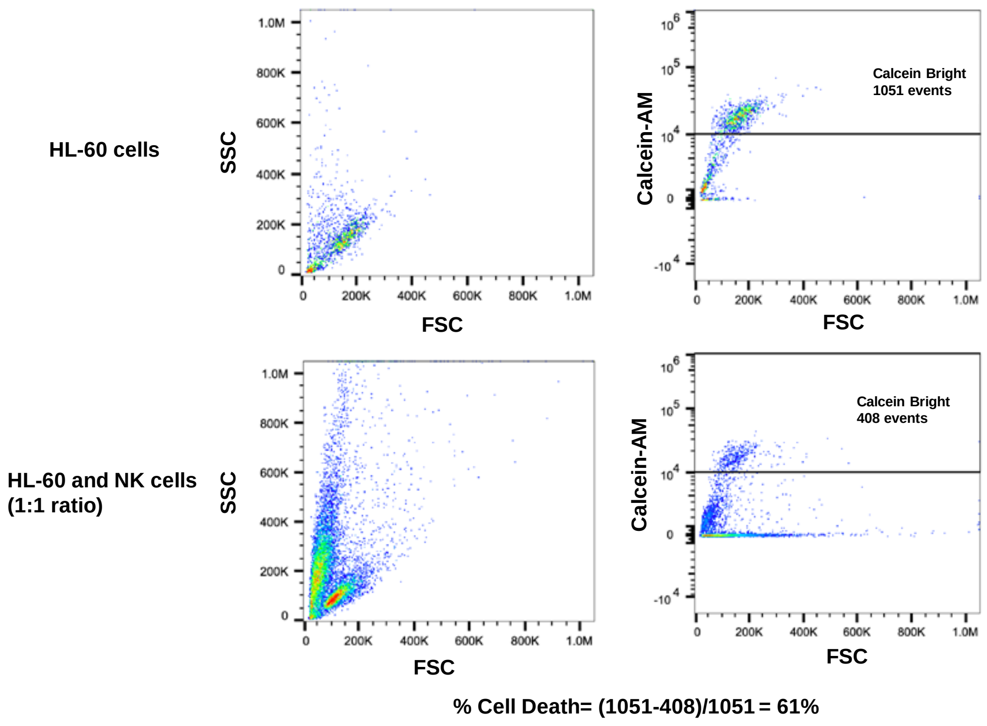

Supplementary Figure 3. Representative example of flow cytometry-based cytotoxicity assay. HL-60 cells were labelled with Calcein AM and then incubated with or without NKF-NK cells at a 1:1 Effector:Target cell ratio for 4 hours.

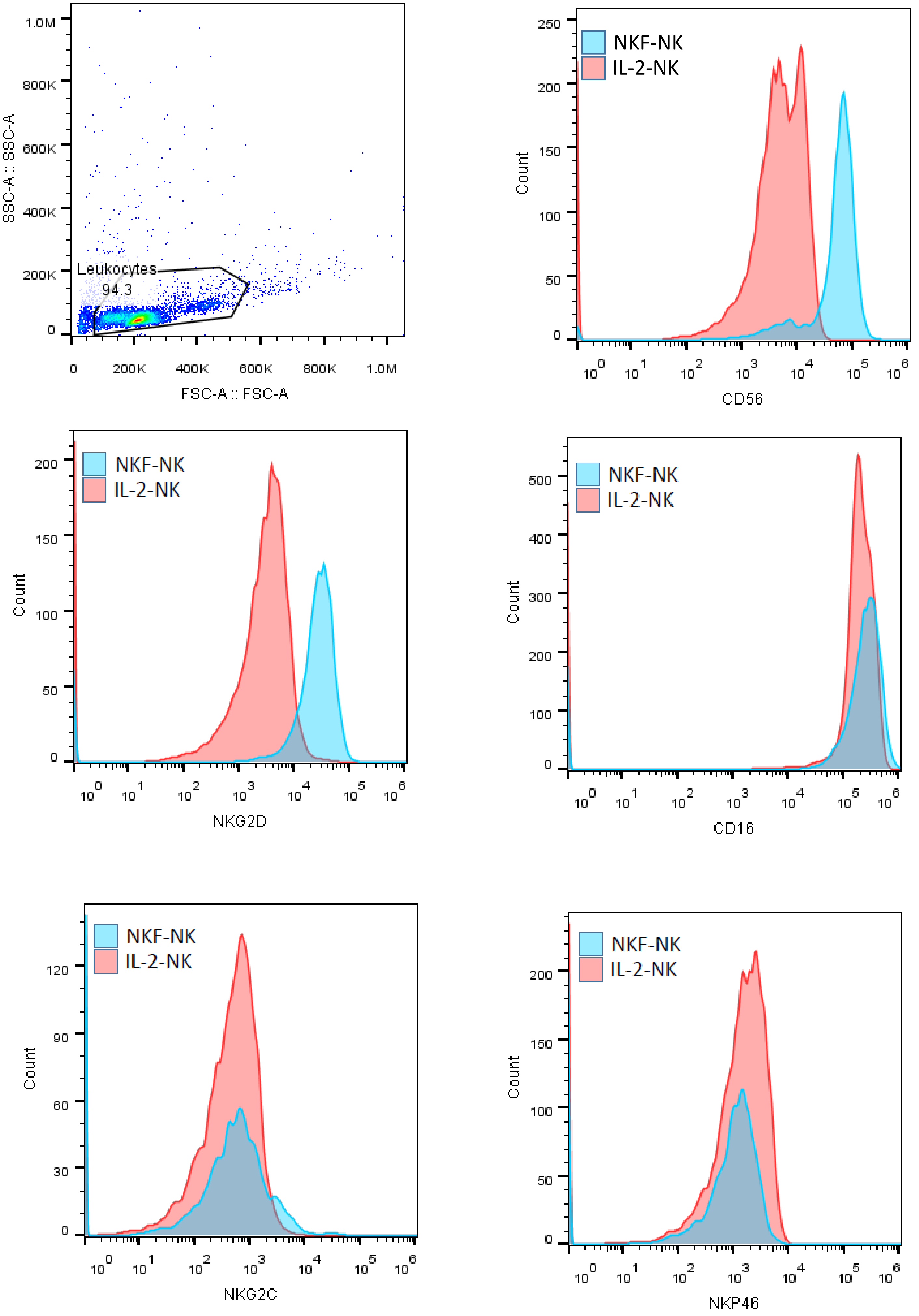

SSC-A :: SSC-A
FSC-A :: FSC-A
Leukocytes
94.3
1.0M
800K
600K
400K
200K
0
200K
400K
600K
800K
1.0M
NKF-NK
IL-2-NK
Count
CD56
NKG2D
CD16
NKG2C
NKP46

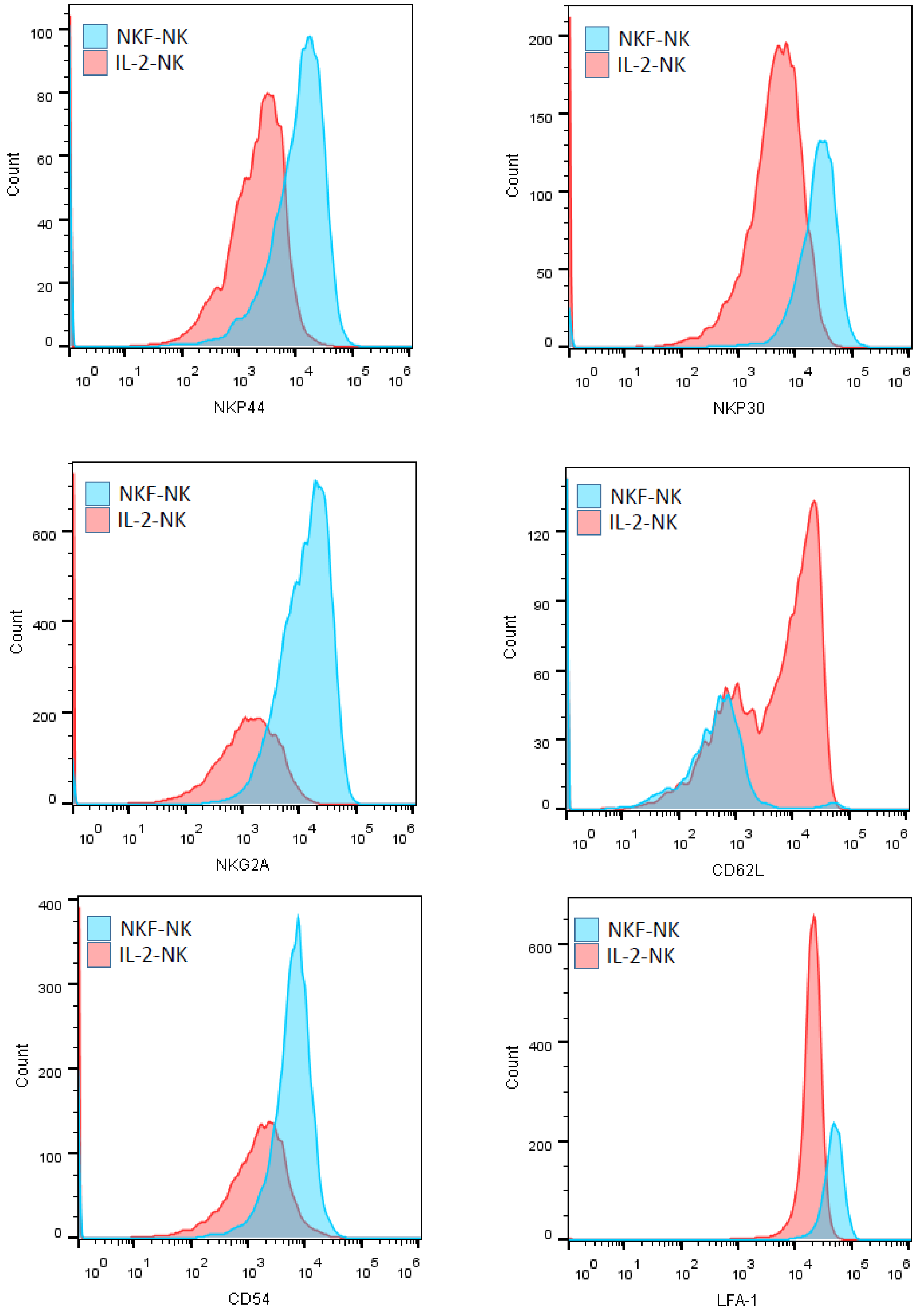
NKF-NK
IL-2-NK
Count
NKP44
NKP30
NKG2A
CD62L
CD54
LFA-1

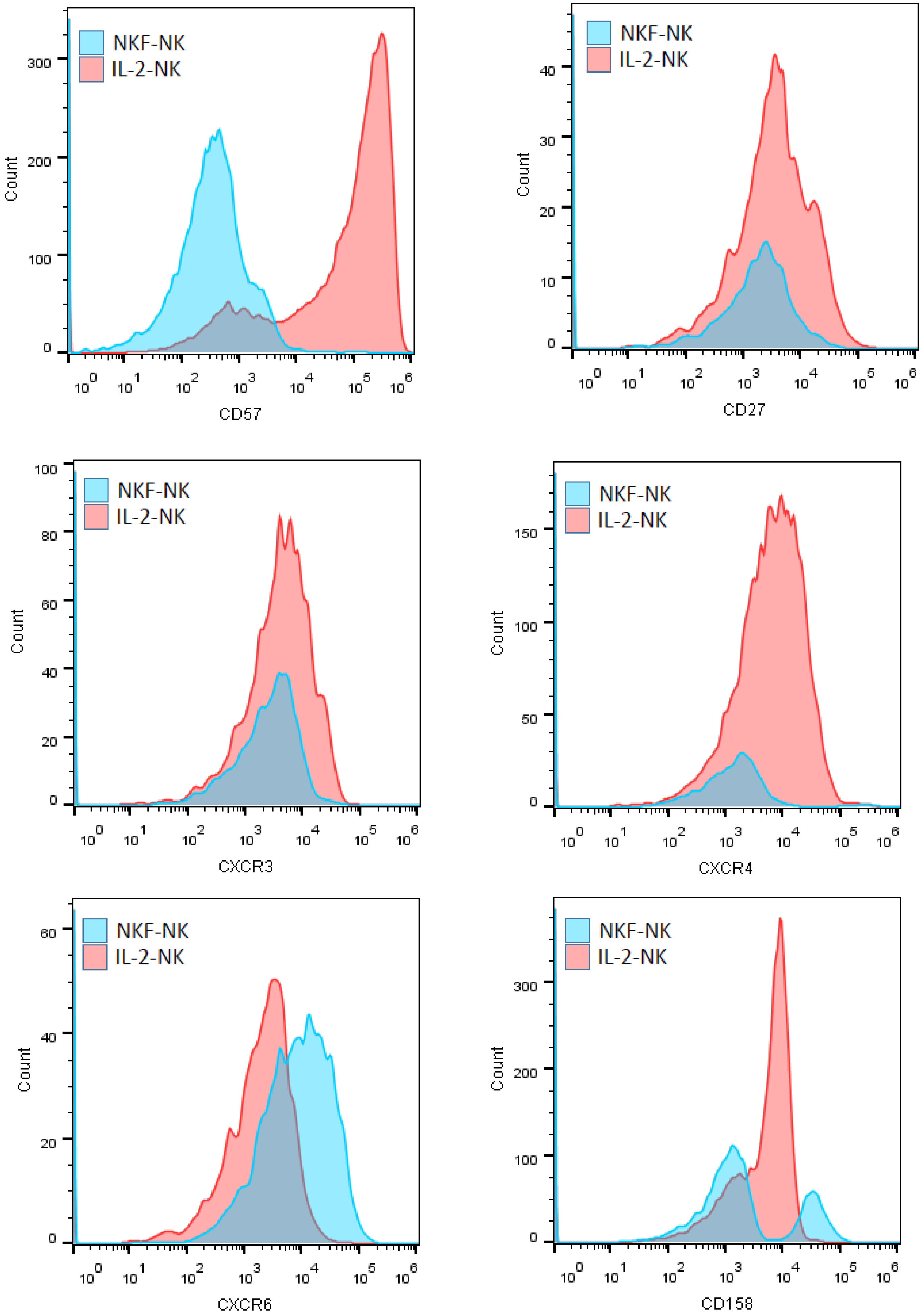

Supplemental Figure 4. Flow cytometry analysis of IL-2-NK and NKF-NK cells for Donor1, including the gating strategy.

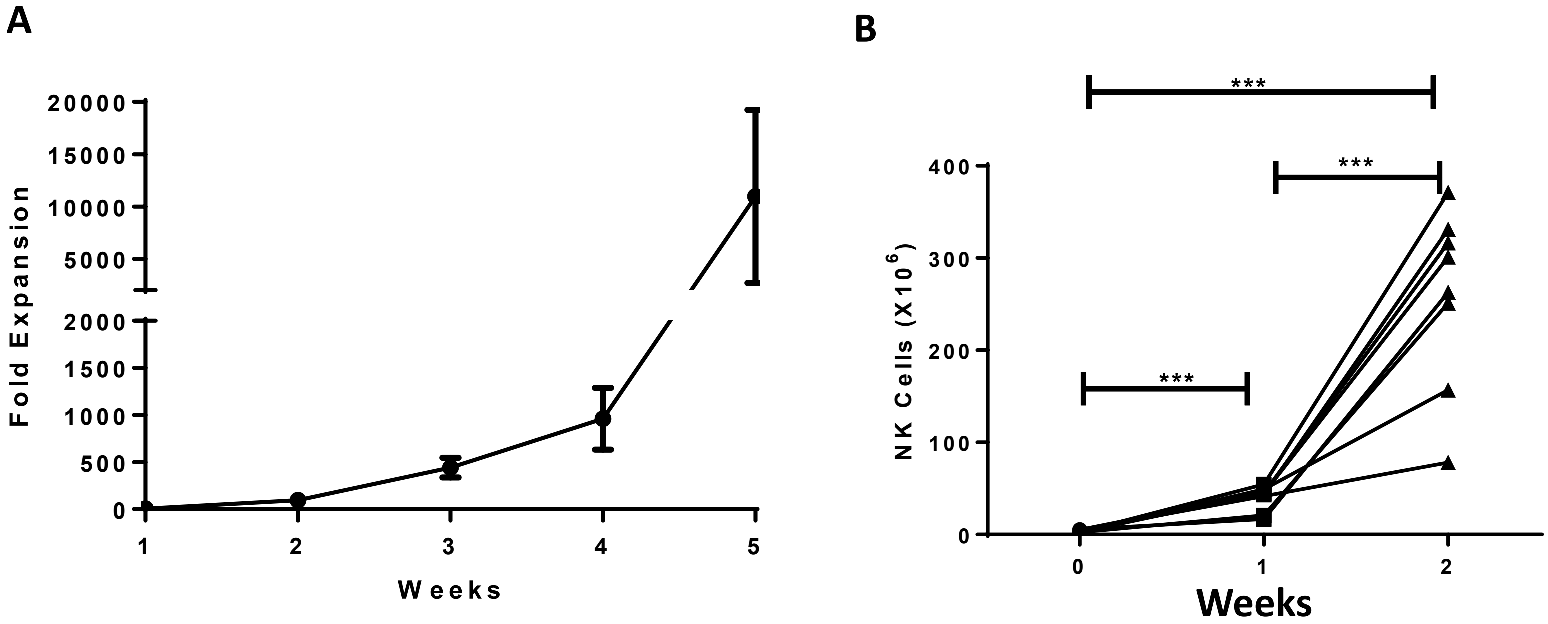

Supplemental Figure 5. A. Fold expansion of NKF-NK cells at 5:1 feeder-to-NK ratio and 200U/mL IL-2 after 5 weeks, n=6. B, Expansion of NK cells using NKF cells at 5:1 feeder-to-NK ratio and 200U/mL IL-2 for 2 weeks, using G-Rex flasks, n=8. ***$p<0.001$. Data represent mean +/- SEM.
